# Digital Economy, Agricultural Technological Progress, and Agricultural Carbon Intensity: Evidence from China

**DOI:** 10.3390/ijerph19116488

**Published:** 2022-05-26

**Authors:** Ruoxi Zhong, Qiang He, Yanbin Qi

**Affiliations:** College of Economics, Sichuan Agricultural University, Chengdu 611130, China; zhongruoxi@stu.sicau.edu.cn (R.Z.); heqiangklcf@stu.sicau.edu.cn (Q.H.)

**Keywords:** digital economy, agricultural carbon intensity, agricultural technological progress, spatial Durbin model

## Abstract

China is the largest carbon emitter in the world, with agricultural carbon emissions accounting for 17% of China’s total carbon emissions. Agricultural carbon emission reduction has become the key to achieving the “Double Carbon” goal. At the same time, the role of the digital economy in achieving the “dual carbon” goal cannot be ignored as an important engine to boost the high-quality development of China’s economy. Therefore, this paper uses the panel data of 30 provinces in mainland China from 2011 to 2019 to construct a spatial Durbin model and a mediation effect model to explore the impact of the digital economy on agricultural carbon intensity and the mediating role of agricultural technological progress. The research results show that: (1) China’s agricultural carbon intensity fluctuated and declined during the study period, but the current agricultural carbon intensity is still at a high level; (2) The inhibitory effect of the digital economy on agricultural carbon intensity is achieved by promoting agricultural technological progress, and the intermediary role of agricultural technological progress has been verified; (3) The digital economy can significantly reduce the carbon intensity of agriculture, and this inhibition has a positive spatial spillover effect. According to the research conclusions, the government should speed up the development of internet technology and digital inclusive finance, support agricultural technology research and improve farmers’ human capital, and strengthen regional cooperation to release the contribution of digital economy space.

## 1. Introduction

At the general debate of the 75th United Nations General Assembly in September 2020, China proposed a “Double Carbon” goal, saying that it would peak carbon dioxide emissions by 2030 and achieve carbon neutrality by 2060 for the first time. As the world’s second-largest economy and a tremendous contributor to the world economy, China has also become a major carbon dioxide emitter [1,2,3]. China’s carbon emissions account for 30% of the world’s total carbon emissions [4,5]. However, China is still in the stage of industrialization and urbanization, so energy consumption is rapidly increasing; this means that China’s carbon dioxide emissions will continue to increase [6,7].

Agriculture is the second-largest source of carbon emissions in the world [8]. According to the IPCC (2014) report, agricultural greenhouse gas emissions account for about 22% of the global total if calculated by 20-year GWP, thus ranking first. If calculated by 100-year GWP, agriculture contributes 14% of global greenhouse gas emissions, second only to industry and electricity and heat production. The total amount of carbon dioxide produced in China’s agricultural production activities accounts for 17% of the country’s total carbon dioxide emissions [2,9]. This puts enormous pressure on the environment [10]. Meanwhile, the deterioration of ecosystem quality caused by environmental pollution may damage food production systems [11]. Therefore, it is urgent to promote agricultural carbon emission reduction.

A broad definition of the digital economy is “the use of ICT in all sectors of the economy” [12]. Based on this, this paper defines the digital economy as an information production factor that uses the Internet as a carrier for economic activities. At present, the digital economy has become the engine of global economic growth and the most active area of China’s economic development [13,14,15]. The “White Paper on China’s Digital Economy Development” (http://www.caict.ac.cn/kxyj/qwfb/bps/202104/t20210423_374626.htm, access date: 28 April 2022) released by the China Academy of Information and Communications Technology in April 2021 shows that the scale of China’s digital economy in 2020 was 39.2 trillion Yuan, an increase of 3.3 trillion Yuan over the preceding year. The digital economy also accounted for 38.6% of Gross Domestic Product, and the growth rate was more than three times that of GDP. The digital economy has become a key driving force for China to stabilize economic growth following the negative impact of COVID-19 and the economic downturn. At the same time, China’s economy has shifted from a high-speed growth stage to a high-quality development stage. As an important starting point for the coordinated development of the economy, environment, and society, the digital economy has attracted widespread attention from scholars. Using panel data of 269 prefecture-level cities in China from 2004 to 2019, Wang et al. found that internet development can promote green economic growth [16]. Usman et al. studied the impact of ICT on economic performance and energy consumption in South Asian economies, arguing that ICT can improve energy efficiency and reduce carbon dioxide emissions [17]. Li, Liu, and Ni [14] used a fixed-effects model to study the impact of the digital economy on carbon dioxide emissions based on the panel data of 190 countries around the world from 2005 to 2016. They found an inverted “U”-shaped relationship between carbon dioxide emissions and the digital economy.

In summary, the inhibitory effect of the digital economy on carbon emissions has been affirmed, but less research has been done on how the digital economy affects the carbon intensity of agriculture and the mechanism behind this effect under the China scenario. Based on this, this paper uses the data of 30 provinces in mainland China from 2011 to 2019 to establish a spatial econometric model and a mediating effect model to investigate the impact of the digital economy on agricultural carbon intensity and the mediating role of agricultural technological progress. The inhibitory effect of the digital economy on carbon intensity is still significant in the agricultural field. The progress of agricultural technology is an important transmission mechanism, and this impact has a positive spillover effect. These conclusions can provide a reference for the Chinese government in achieving agricultural carbon emission reduction. This paper may have the following two innovations: (1) Discussion of the impact of the digital economy on agricultural carbon intensity, and expansion of the research and existing literature in this regard, and (2) The digital economy, agricultural technological progress, and agricultural carbon intensity are brought into the same research framework, and the path through which the digital economy affects agricultural carbon intensity is examined. It supports the conjecture that the progress of agricultural technology plays an intermediary role in the effect of the digital economy on reducing the carbon intensity of agriculture and helps to clarify the path of the effect of the digital economy on the carbon intensity of agriculture.

The rest of this paper is set up as follows: Section 2 briefly introduces the research hypothesis. Section 3 measures China’s agricultural carbon intensity and explains the estimation methods and data used in this paper. Section 4 presents the empirical results and discussion. Section 5 provides conclusions and policy recommendations.

## 2. Theoretical Analysis and Research Assumptions

**Figure 1:**
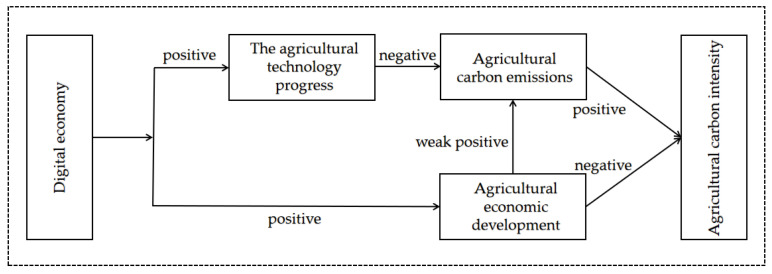
Mechanism path chart of the digital economy as it affects agricultural carbon intensity.

From the formula of agricultural carbon intensity, reducing agricultural carbon emissions and developing the agricultural economy will reduce agricultural carbon intensity. The effect of the digital economy on agricultural carbon intensity can be achieved by affecting agricultural carbon emissions and agricultural economic development, as shown in Figure 1.

### 2.1. Digital Economy and Agricultural Carbon Intensity

The digital economy can promote the development of the agricultural economy and reduce the carbon intensity of agriculture. Some scholars state that the development of the internet and inclusive finance can increase farmers’ incomes and promote agricultural economic growth [18,19,20], which will lead to an increase in agricultural carbon emissions [10,21,22]. From the formula of agricultural carbon intensity, agricultural economic development can, on the one hand, reduce agricultural carbon intensity, and on the other hand, will promote agricultural carbon emissions and increase agricultural carbon intensity. Therefore, the impact of the digital economy on agricultural carbon intensity by promoting agricultural economic growth will result in both “increase” and “decrease” effects. Therefore, what kind of results will China’s digital economy achieve by promoting the development of the agricultural economy to affect the carbon intensity of agriculture? This paper argues that China’s digital economy will reduce agricultural carbon intensity by promoting agricultural economic development. Because Chinese leaders pay more attention to green development, a strict environmental protection system and official evaluation methods, including ecological indicators, will force local governments to give up the GDP-based development method used exclusively in the past. They will have to consider how to realize the coordinated development of the economy and environment [23]. Therefore, the role of agricultural economic development in promoting carbon emissions will gradually fail.

The digital economy can promote the advancement of agricultural technology through information transfer and relaxation of loan restrictions. First, the digital economy provides farmers with an information exchange platform and reduces the marginal cost of their interaction. Through the internet, mobile phones, and other media, farmers can obtain the technical information needed for production activities, and then share the information with other farmers [24]. Secondly, the learning and utilization of new technologies and the transformation of technological achievements are inseparable from financial support [25]. Digital inclusive finance based on the development of information technology has effectively lowered the threshold of financial services and broadened the scope of financial services. It can also provide financial support for farmers to implement agricultural technology by reducing credit restrictions [26], while promoting the application of new technologies in agricultural production.

At the same time, the progress of agricultural technology can effectively reduce agricultural carbon emissions [10,27]. First, the progress of agricultural technology can improve the utilization efficiency of agricultural production factors [10,28], obtain a given output with less input, and reduce agricultural carbon emissions caused by the expansion of the agricultural production scale. Second, the progress of agricultural technology can improve the effectiveness of agricultural pollution control. After production factors such as pesticides, fertilizers, and agricultural films are used, harmful substances will remain in the land [29], and these harmful substances will be reduced to carbonitrides and cause greenhouse gas emissions. Technological progress in agricultural pollution control can reduce harmful substances caused by agricultural production factors [28], and reduce agricultural carbon emissions. Therefore, the progress of agricultural technology can play a role in two stages—before and after agricultural carbon emissions, and effectively reduce agricultural carbon emissions. Based on this, we propose Hypothesis 1 and Hypothesis 2:

**Hypothesis** **1.** *The digital economy has an inhibitory effect on agricultural carbon intensity.*

**Hypothesis** **2.** *Digital economy can reduce agricultural carbon intensity by boosting agricultural technology*.

### 2.2. The Impact of the Digital Economy on Agricultural Carbon Intensity Has Spatial Spillover Effects

Yilmaz, et al. [30] were among the first to pay attention to the spatial spillover effect of the digital economy, using the panel data of 48 states in the United States from 1970 to 1997 to test the spatial spillover effect of state-level telecommunications infrastructure investment on national output. After that, Zhou et al. [31], Wu et al. [32], and Su et al. [33] found that the impact of digital finance and the Internet on green development and ecological efficiency has a spatial spillover effect. He et al. [34] used data from 31 provinces in China from 2007 to 2017 to determine that agricultural greenhouse gases are spatially autocorrelated, and agricultural technological progress has a spatial spillover effect on the reduction of greenhouse gas emissions. Due to the mobility of greenhouse gases such as carbon dioxide and the geographical connection of each region [35], agricultural carbon emissions in a region will affect adjacent regions. Thus, agricultural carbon intensity has spatial autocorrelation. Local experience and achievements in digital economy development can flow into surrounding areas through regional cooperation, improving the level of the digital economy in adjacent areas. On the one hand, it can, in this way, improve agricultural production efficiency in adjacent areas, promote agricultural economic growth, and reduce agricultural carbon intensity. On the other hand, it can promote the diffusion of agricultural technology between regions, improve the level of agricultural technology in adjacent regions, and reduce agricultural carbon intensity. Based on this, we propose Hypothesis 3:

**Hypothesis** **3.** *The digital economy can affect agricultural carbon intensity in adjacent areas through spatial spillover effects.*

## 3. Research Design

### 3.1. Calculation of Agricultural Carbon Emissions and Agricultural Carbon Intensity

#### 3.1.1. Calculation of Agricultural Carbon Emissions

This paper takes agriculture as the research object in a narrow sense (planting industry). According to previous research, the carbon sources of the planting industry mainly include fertilizer, pesticide, agricultural film, irrigation, ploughing, machinery, and diesel oil [22,36,37,38]. Therefore, the agricultural carbon emissions measured in this paper include these seven aspects. Drawing on the method of Huang, Xu, Wang, Zhang, Gao and Chen [36,37,38], the following formula for calculating agricultural carbon emissions is constructed:

(1) $$E=\sum_{i=1}^{\;n}E_{i}=\sum_{i=1}^{\;n\;}C_{i}{\times \delta }_{i}$$

where n is the number of agricultural input elements (carbon sources); E is the total amount of agricultural carbon emissions, which is equal to the sum of carbon emissions from various carbon sources; E_i_ is the carbon emissions of the i-th agricultural input element, including fertilizers, pesticide, agricultural film, irrigation, farming, machinery, diesel oil; C_i_ is the amount of the i-th agricultural input element; and δ_i_ is the carbon emission coefficient of the i-th agricultural input element (Table 1).

#### 3.1.2. Calculation of Agricultural Carbon Intensity

This paper draws on the method of Zhou*,* et al. [40] to construct the following formula for calculating agricultural carbon intensity:

(2) ACI=E/AV

where ACI is the agricultural carbon intensity, E is the agricultural carbon emissions calculated by Equation (1), and AV is the added value of the primary industry.

### 3.2. Benchmark Regression Model

#### 3.2.1. Basic Model

To verify H1, the following panel regression model is established:

(3) $${\mathrm{ACI}}_{\mathrm{it}}=\partial_{0}+\partial_{1}{\mathrm{DIG}}_{\mathrm{it}}+\partial_{2}{\mathrm{LnUR}}_{\mathrm{it}}+\partial_{3}{\mathrm{LnER}}_{\mathrm{it}}+\partial_{4}{\mathrm{LnSTRU}}_{\mathrm{it}}+\partial_{5}{\mathrm{LnRTI}}_{\mathrm{it}}+\partial_{6}{\mathrm{LnAFFI}}_{\mathrm{it}}+\partial_{7}{\mathrm{LnAFE}}_{\mathrm{it}}\mu_{i}{+\lambda }_{t}{+\varepsilon }_{\mathrm{it}}$$

where ${\mathrm{ACI}}_{\mathrm{it}}$ is the agricultural carbon intensity of city i in year t, with i = 1, 2, …, 30; t = 2011, 2012, …, 2019; $\partial_{0}$ is the intercept; $\partial_{n}$ (n = 1, 2, …, 6, 7) are the coefficients of the variables; DIG_it_ is the digital economy of city i in year t; LnUR_it_ is the urbanization rate of city i in year t; LnER_it_ is the environmental regulation of city i in year t; LnSTRU_it_ is the industrial structure of city i in year t; LnRTI_it_ is the road traffic infrastructure of city i in year t; LnAFFI_it_ is the disaster rate of city i in year t; LnAFE_it_ is the agricultural fiscal expenditure of city i in year t; μ_i_ is the individual fixed effects; λ_t_ is the time fixed effects; and ε_it_ is the random disturbance term.

#### 3.2.2. Mediation Effect Model

To identify the possible mechanism of action of the digital economy on agricultural carbon intensity, and to test whether agricultural technological progress is an intermediary variable between the two, the following intermediary model was established based on the experience of Wang*,* et al. [41]. First, on the premise that the coefficient $\partial_{1}$ in Equation (3) is significant, the regression Equation (4) of the digital economy and agricultural carbon intensity is established. Then, the regression Equation (5) of the digital economy, agricultural technological progress, and agricultural carbon intensity is established, and the existence of the mediating effect is judged according to the magnitude and significance of the coefficients $\rho_{1}$, $\varphi_{1}$ and $\varphi_{2}$:

(4) $${\mathrm{TE}}_{\mathrm{it}}{=\rho }_{0}{+\rho }_{1}{\mathrm{DIG}}_{\mathrm{it}}{+\rho }_{2}{\mathrm{LnUR}}_{\mathrm{it}}{+\rho }_{3}{\mathrm{LnER}}_{\mathrm{it}}{+\rho }_{4}{\mathrm{LnSTRU}}_{\mathrm{it}}{+\rho }_{5}{\mathrm{LnRTI}}_{\mathrm{it}}{+\rho }_{6}{\mathrm{LnAFFI}}_{\mathrm{it}}{+\rho }_{7}{\mathrm{LnAFE}}_{\mathrm{it}}\mu_{i}{+\lambda }_{t}{+\varepsilon }_{\mathrm{it}}$$

(5) $${\mathrm{ACI}}_{\mathrm{it}}{=\varphi }_{0}{+\varphi }_{1}{\mathrm{DIG}}_{\mathrm{it}}{+\varphi }_{2}{\mathrm{TE}}_{\mathrm{it}}{+\varphi }_{3}{\mathrm{UR}}_{\mathrm{it}}{+\varphi }_{4}{\mathrm{ER}}_{\mathrm{it}}{+\varphi }_{5}{\mathrm{STRU}}_{\mathrm{it}}{+\varphi }_{6}{\mathrm{RTI}}_{\mathrm{it}}{+\varphi }_{7}{\mathrm{AFFI}}_{\mathrm{it}}{+\varphi }_{8}{\mathrm{LnAFE}}_{\mathrm{it}}{+\mu }_{i}{+\lambda }_{t}{+\varepsilon }_{\mathrm{it}}$$

where TE_it_ is the intermediary variable agricultural technology progress of city i in year t, and other variables and symbols are consistent with Formula (3).

### 3.3. Spatial Autocorrelation Calculation of Agricultural Carbon Intensity

#### 3.3.1. Global Moran’s I

It is necessary to test whether the research object has spatial effects before the analysis. Global Moran’s I is a derivation of Moran’s I with a value range of [−1, 1]. When the global Moran’s I is greater than 0, the data is positively correlated in space, and the closer it is to 1, the stronger the positive correlation. A global Moran’s I less than 0 means that the data is negatively correlated in space, and the closer it is to −1, the stronger the negative correlation. When the global Moran’s I is equal to 0, the data has no spatial autocorrelation. The formula for the global Moran’s I is as follows

(6) $$I=\frac{n}{\sum_{i=1}^{n}\sum_{j=1}^{n}W_{\mathrm{ij}}}\times \frac{\sum_{i=1}^{n}\sum_{j=1}^{n}W_{\mathrm{ij}}\left({\mathrm{ACI}}_{i}-\bar{\mathrm{ACI}}\right)\left({\mathrm{ACI}}_{j}-\bar{\mathrm{ACI}}\right)}{\sum_{i=1}^{n}{\left({\mathrm{ACI}}_{i}\;-\;\bar{\mathrm{ACI}}\right)}^{2}}$$

where I is the global Moran’s I; n is the number of observations; W_ij_ is the geographic distance weight matrix; ACI_i_ and ACI_j_ are the agricultural carbon intensity in regions i and j; and $\bar{\mathrm{ACI}}$ is the mean of all observations of ACI.

#### 3.3.2. Local Moran’s I

To analyze the scope and location of agricultural carbon intensity in spatial agglomeration, this paper uses the local Moran’s I to reflect the spatial autocorrelation of agricultural carbon intensity. The formula is as follows:

(7) $$I_{i}=\frac{n\left({\mathrm{ACI}}_{i}\;\;-\;\bar{\mathrm{ACI}}\right)}{\sum_{i=1}^{n}{\left({\mathrm{ACI}}_{i}\;\;-\;\bar{\;\mathrm{ACI}}\right)}^{2}}\sum_{i=1,\;j\neq i}^{n}W_{\mathrm{ij}}\left({\mathrm{ACI}}_{j}\;-\;\bar{\mathrm{ACI}}\right)$$

where I_i_ is the local Moran’s I; and the definitions of n, W_ij_, ACI_i_, ACI_j_, and $\bar{\mathrm{ACI}}$ are the same as those of Formula (6). When I_i_ is greater than 0, the agricultural carbon intensity in the i region is similar to the adjacent areas. When I_i_ is less than 0, the agricultural carbon intensity in the i region is significantly different from the adjacent areas.

### 3.4. Spatial Durbin Model

To verify H3, the following spatial SDM model is constructed:

(8) $$\begin{array}{c} {\mathrm{ACI}}_{\mathrm{it}}{=\beta }_{0}{+\beta }_{1}{\mathrm{DIG}}_{\mathrm{it}}{+\beta }_{2}{\mathrm{TE}}_{\mathrm{it}}{+\beta }_{3}{\mathrm{LnUR}}_{\mathrm{it}}{+\beta }_{4}{\mathrm{LnER}}_{\mathrm{it}}{+\beta }_{5}{\mathrm{LnSTRU}}_{\mathrm{it}}{+\beta }_{6}{\mathrm{LnRTI}}_{\mathrm{it}}{+\beta }_{7}{\mathrm{LnAFFI}}_{\mathrm{it}}{+\beta }_{8}{\mathrm{LnAFE}}_{\mathrm{it}}{+\rho \mathrm{WACI}}_{\mathrm{it}}{+\varphi }_{1}\\ {\mathrm{WDIG}}_{\mathrm{it}}\;{+\varphi }_{2}{\mathrm{WTE}}_{\mathrm{it}}{+\varphi }_{3}{\mathrm{WLnUR}}_{\mathrm{it}}{+\varphi }_{4}{\mathrm{WLnER}}_{\mathrm{it}}{+\varphi }_{5}{\mathrm{WLnSTRU}}_{\mathrm{it}}{+\varphi }_{6}{\mathrm{WLnRTI}}_{\mathrm{it}}{+\varphi }_{7}{\mathrm{WLnAFFI}}_{\mathrm{it}}{+\varphi }_{8}{\mathrm{WLnAFE}}_{\mathrm{it}}{+\mu }_{i}{+\lambda }_{t}{+\varepsilon }_{\mathrm{it}} \end{array}$$

where $\beta_{0}$ is the intercept; $\beta_{n}$(n = 1, 2, …, 7, 8) are the coefficients of the variables; ρ is the spatial autoregressive coefficient of the dependent variable; W is the geographic distance weight matrix; $\varphi_{n}$(n = 1, 2, …, 7, 8) are the spatial spillover coefficients of the digital economy and control variables; and other variables and symbols are the same as Formula (3).

### 3.5. Variable Selection and Data Sources

#### 3.5.1. Core Explanatory Variable

According to research by Bukht and Heeks [12] and Du and Guan [42], this paper defines the digital economy as the information production factors that use the Internet as a carrier of economic activities. Referring to the methods of Huang et al. [43] and Zhao, Zhang, and Liang [13], a comprehensive index of the digital economy, including the Internet and digital financial inclusion, is constructed. First, the index score is calculated using the entropy value method, and then the standardized index value is multiplied by the score to obtain the comprehensive index of the digital economy. The specific indicators and scores are shown in Table 2. The level of Internet development from the two aspects of use and output are measured, and four indicators, i.e., internet penetration rate, internet-related employees, internet-related output, and the number of mobile internet users, are selected. The internet penetration rate is represented by the number of internet users per 100 people; the internet-related employees are represented by the proportion of employees in computer service and software industries in the unit employees; the internet-related output is represented by the total number of telecommunication services per capita; and the number of mobile internet users is represented by mobile phone users per 100 people. The Digital Finance Research Center of Peking University uses the micro data of Ant Financial, a representative Internet financial institution in China, on digital inclusive finance, and constructs the digital inclusive financial index from three aspects: depth of use, breadth of coverage, and digital support services [44]. This paper uses this index to characterize the level of digital financial development, thus reflecting the reachability and service scope of inclusive finance in China’s provinces.

#### 3.5.2. Mediating Variable

The progress of agricultural technology is represented by the number of patents authorized in the agricultural field per capita in the primary industry. The calculation of the number of patents granted in the agricultural field draws on the method of Liu, Ji, Zhang, An, and Sun [26], and is represented by the sum of the number of invention patents and utility model patents in the agricultural field obtained from the CNKI (China National Knowledge Infrastructure: https://www.cnki.net/, access date: 28 April 2022) patent database.

#### 3.5.3. Control Variables

In the research on the influencing factors of agricultural carbon emissions, the selection of control variables mainly involves four aspects: (1) the level of agricultural economy [21]; (2) industrial structure [45]; (3) population structure and agricultural disaster situation [46]; and (4) agricultural financial support [47]. In addition, some scholars state that transportation and the environment also have an impact on carbon emissions [48,49,50]. Drawing on past research and combining data availability, this paper selects urbanization, industrial structure, agricultural disaster rate, agricultural fiscal expenditure, road traffic infrastructure, and environmental regulation as control variables. The urbanization level is characterized by the ratio of the urban population to the total population [51]; the industrial structure is characterized by the ratio of the added value of non-agricultural industries to GDP [27]; the agricultural disaster rate is characterized by the ratio of the affected agricultural area to the total sown area [52]; the agricultural fiscal expenditure is characterized by the ratio of expenditure on agriculture, forestry, and water affairs to the total expenditure of government fiscal final accounts [47]; the transportation infrastructure is characterized by road miles [53]; and the environmental regulation is characterized by the ratio of the investment in environmental pollution control to GDP [54].

The meanings, symbols, and units of these variables are shown in Table 3.

#### 3.5.4. Data Sources

This paper takes 30 provinces in inland China (excluding Tibet) as the research object, and the time range is 2011 to 2019.

Digital financial inclusion data is from Guo et al. [44]. The data on internet indicators, industrial structure, added value of the primary industry, number of employed persons in the primary industry, expenditure on agriculture, forestry and water affairs, and road mileage are from China Statistical Yearbook. The data on environmental regulation is from China Environmental Statistical Yearbook. The data on the affected area of land, the total sown area, and the seven agricultural input factors are from the China Rural Statistical Yearbook. The data on urbanization is from the China Regional Statistical Yearbook. The missing values are filled with the average growth rate. To avoid heteroscedasticity, some indicators are processed by logarithm. To mitigate the impact of inflation, some indicators are deflated with 2011 as the base year and adjusted to constant prices. The descriptive statistics of the variables are shown in Table 4.

## 4. Empirical Results and Analysis

### 4.1. Status Quo Analysis of Agricultural Carbon Intensity, Digital Economy and Agricultural Technology Progress

**Figure 2:**
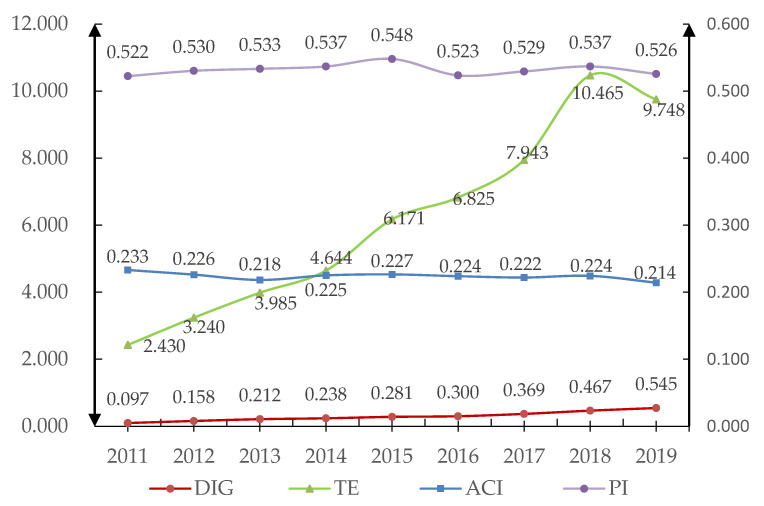
Time evolution diagram of China’s agricultural carbon emissions, digital economy, agricultural technology progress, and the proportion of crop production value.

This paper plots the temporal evolution of agricultural carbon intensity, the digital economy, and agricultural technological progress from 2011 to 2019 at the national level. In Figure 2, the left *y*-axis in the range of [0.000, 12.000] is the vertical axis of the digital economy and agricultural technology progress. The right *y*-axis in the range of [0.200, 0.235] is the vertical axis of the agricultural carbon intensity and the proportion of crop production value, while the horizontal axis represents the year.

The fluctuating decline of China’s agricultural carbon intensity from 2011 to 2019 shows that China’s past efforts to reduce agricultural carbon emissions have paid off. However, China’s agriculture still faces huge challenges. Large-scale agricultural production still leads to a high amount of agricultural carbon intensity. Ensuring food security while considering the environment is the most important challenge that China must overcome at present [55].

China’s agricultural technology progress shows an overall increasing trend from 2011 to 2019. The number of agricultural patents per capita in the primary industry increased from 2.43 to 9.75, an increase of 301.18%, indicating that China’s agricultural technology research and development made great progress. However, China’s current level of agricultural technology is relatively low, and agricultural technology presents the characteristics of “high energy consumption and high emissions” [56], so it cannot greatly reduce agricultural carbon intensity. In the future, it will be necessary to further increase investment in technology research and development in the agricultural field and focus on agricultural technology with the characteristics of “improving efficiency and controlling pollution” to promote carbon emission reduction in agriculture.

The digital economy continued to improve from 2011 to 2019, indicating that the promotion of digital China has achieved results. The growth rate of the digital economy increased even more after China released “The Thirteen Five-Year Plan for National Economic and Social Development of the People’s Republic of China” in March 2016. It officially proposed to combine information technology and economic and social development to promote the development of the digital economy, providing a strong policy guarantee for the rapid development of the digital economy. Among them, the digital economy level in the eastern region is the highest, while that of the western region is the lowest. According to the “China Regional and Urban Digital Economy Development Report (2020)” released by the China Academy of Information and Communications Technology (http://www.caict.ac.cn/kxyj/qwfb/ztbg/202101/t20210104_367593.htm, access date: 28 April 2022), the eastern region accounted for eight out of the top 15 provinces in China’s digital economy competitiveness in 2019, with Guangdong, Beijing, and Shanghai ranking among the top three. Meanwhile, the central region accounted for four, and the western region accounted for only three. As the frontier of China’s reform and innovation, the eastern region had the opportunity to develop the digital economy earlier and had more technology and funds to support the development of the digital economy, eventually becoming the leading region in China’s digital economy.

The proportion of China’s planting industry output value to the total output value of agriculture, forestry, animal husbandry, and fishery fluctuated slightly from 2011 to 2019 but has remained at a high level of 52%. The stable development of the planting industry is of great significance to improving people’s living standards and ensuring national food security.

The Chinese government has divided its regions into the main grain producing area, the main grain sales area, and the production and sales balance area (see Table A2 of Appendix A for details). Among them, the main sales areas are concentrated in the east, and their average self-sufficiency rate for food is less than 30% (Data source: Du Ying: “China’s Food Security Strategy (Part 2)”, “China Rural News Agency”, No. 22, 2020.). Their contribution to national agriculture is very small, indicating a negative correlation between the digital economy and the development level of the planting industry. While undertaking the important task of ensuring national food security, the main grain-producing areas also face various natural, market and policy risks, creating the dilemma of “the provinces with large grains are often economically weak and financially poor” [57]. According to the research of the “White Paper on China’s Digital Economy Development” (http://www.caict.ac.cn/kxyj/qwfb/bps/202104/t20210423_374626.htm, access date: 28 April 2022), China’s digital economy has a strong positive correlation with the level of national economic development, and the limited regional economic development has further led to a low level of regional digital economy development.

### 4.2. Benchmark Regression and Mediation Effect Results Analysis

Columns (1) and (2) of Table 5 report the results of the panel benchmark regression. Whether the control variable is added or not, the digital economy can significantly reduce agricultural carbon intensity, and H1 is verified. After adding the control variables, the coefficient of the digital economy is −0.250, indicating that increasing the digital economy by one unit can reduce the agricultural carbon intensity by 0.250 units. The industrial structure, agricultural disaster rate, and agricultural fiscal expenditure will significantly increase the carbon intensity of agriculture. The industrial structure can significantly increase the carbon intensity of agriculture because the proportion of non-agricultural industries in GDP increases, and then more resources will be tilted towards non-agricultural industries, thus increasing the carbon intensity of agriculture. According to the research of He et al. [58], land damage will affect the final output, but the input of production factors in the early stage will not reduce agricultural carbon emissions, which will lead to an increase in agricultural carbon intensity. Agricultural fiscal expenditure will expand the scale of agricultural production, which is consistent with the research of Wang and Li [59]. This may be because agricultural fiscal expenditures have increased the input of production factors such as fertilizers and pesticides, thus increasing agricultural carbon intensity. The remaining control variables have no significant effect on agricultural carbon intensity.

Columns (3) and (4) of Table 5 report the results of the mediation test. Under the premise that the digital economy coefficient in column (2) is significantly negative, the results in column (3) show that the digital economy can significantly promote the progress of agricultural technology. The results in column (3) show that the digital economy can significantly promote technological progress in agriculture, and the results in column (4) show that technological progress in agriculture can significantly reduce agricultural carbon intensity. Compared with column (2), the coefficient of the digital economy in column (4) is smaller, indicating that the digital economy’s inhibitory effect on the carbon intensity of agriculture is partly achieved through the advancement of agricultural technology. H2 is verified.

### 4.3. Spatial Autocorrelation Results of Agricultural Carbon Intensity

#### 4.3.1. Global Moran’s I Analysis

Based on the geographic distance weight matrix, this paper measures the global Moran index of agricultural carbon intensity from 2011 to 2019. Table 6 shows the global Moran index of agricultural carbon intensity from 2011 to 2019 to be greater than 0. Also, there are several years of agricultural carbon intensity with significant positive autocorrelation in space, which may be because agricultural production is greatly affected by natural conditions. Adjacent regions have similar production conditions and production methods, resulting in similar agricultural carbon intensities. Therefore, a spatial model can be introduced when exploring the impact of the digital economy on China’s agricultural carbon intensity [60].

#### 4.3.2. Local Moran’s I Analysis

**Figure 3:**
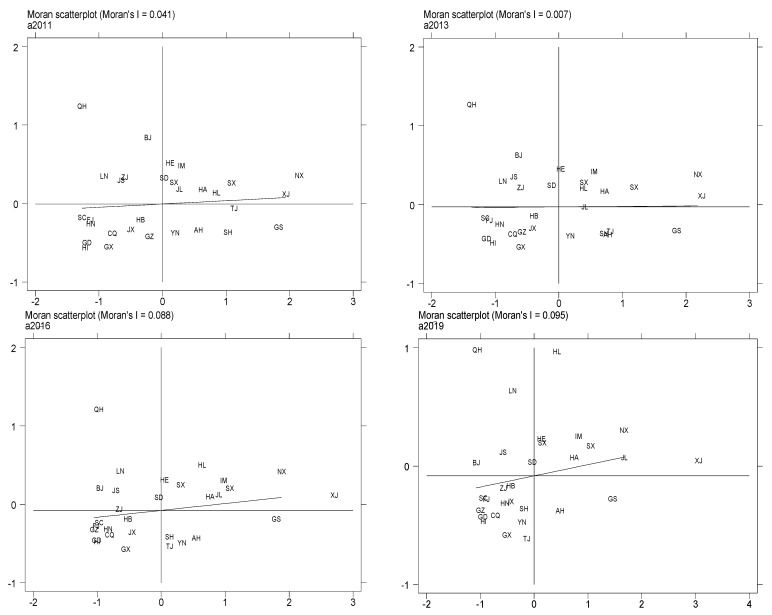
Moran scatter plots of agricultural carbon intensity in China for 2011, 2013, 2016, and 2019.

Figure 3 presents a partial Moran scatter plot of agricultural carbon intensity in 2011, 2013, 2016, and 2019. The letters in the plot are the abbreviations of the names of the provinces in China (see Table A1 of Appendix A for details). The abscissa of Moran’s scatter plot represents the normalized agricultural carbon intensity, and the ordinate is the spatial lag term of agricultural carbon intensity. Among the four areas divided by the two coordinate axes, the upper right area is the first quadrant, the upper left area is the second quadrant, the lower left area is the third quadrant, and the lower right area is the fourth quadrant. The first quadrant represents high–high (HH) aggregation, representing provinces with high agricultural carbon intensity with corresponding high agricultural carbon intensity in their surrounding provinces. The second quadrant represents low–high (LH) aggregation, representing provinces with low agricultural carbon intensity that have high agricultural carbon intensity in their surrounding provinces. The third quadrant represents low–low (LL) aggregation, representing provinces with low agricultural carbon intensity with corresponding low agricultural carbon intensity in their surrounding provinces. The fourth quadrant represents high–low (HL) aggregation, representing provinces with high agricultural carbon intensity that have low agricultural carbon intensity in their surrounding provinces. Figure 3 shows that while the positions of individual cities on the Moran scatter diagram have changed, there are more provinces in the first and third quadrants than in the second and fourth quadrants, confirming the previous conclusion that China’s agricultural carbon intensity has a positive spatial autocorrelation.

### 4.4. Analysis of Spatial Spillover Effects

#### 4.4.1. Choice of Spatial Model

According to Elhorst [61], several tests need to be carried out to select the most suitable spatial econometric model before performing spatial econometric regression. Based on the geographic distance weight matrix, LM, Wald, LR, Hausman, and fixed effects tests were performed, and the results are shown in Table 7. The test results of LM-LAG, LM-ERR, Robust LM-LAG, and Robust LM-ERR passed the significance test, indicating that a spatial econometric model should be used. Secondly, the test results of Wald-SAR, Wald-SEM, LR-SAR, and LR-SEM also passed the significance test, rejecting the null hypothesis that the SDM model can be degenerated into a SAR model or a SEM model, and indicating that the SDM model should be introduced. Finally, the Hausman test results show that the fixed effects model is better than the random effects model, indicating that the fixed effects SDM model should be established based on the geographic distance weight matrix.

#### 4.4.2. Analysis of SDM Regression Results

According to the choice of the spatial econometric model, a fixed effects SDM model based on the spatial geographic distance weight matrix is established for regression. The results in Table 8 show that the digital economy has a significant inhibitory effect on the local agricultural carbon intensity. It also shows that the industrial structure, agricultural disaster rate, and agricultural financial expenditure significantly affect the local agricultural carbon intensity. The digital economy and industrial structure have a significant inhibitory effect on the agricultural carbon intensity of adjacent areas, while the agricultural disaster rate and agricultural fiscal expenditure have a significant effect on the agricultural carbon intensity of adjacent areas. As direct analysis of SDM estimates may lead to incorrect conclusions [62], this paper next discusses the direct and indirect effects of independent variables.

#### 4.4.3. Analysis of Direct and Indirect Effects

Table 9 presents the effect of independent variables on agricultural carbon intensity. Direct effect refers to the influence of the independent variable on its own agricultural carbon intensity, while the indirect effect of the independent variable is its influence on the agricultural carbon intensity of adjacent areas. The sum of the two is the total effect.

From the regression results in Table 9, the direct effect of the digital economy on agricultural carbon intensity is −0.200, which is significant at the 1% level. For each unit of increase in the digital economy, agricultural carbon intensity will decrease by 0.2%. The direct effect coefficient of industrial structure on agricultural carbon intensity is 0.777, which is significant at the 1% level. For each unit of industrial structure increase, agricultural carbon intensity will increase by 0.777%. The added value of non-agricultural industries accounts for an increase in the proportion of GDP, and more labor is transferred from agriculture to non-agricultural industries. The reduction of agricultural labor is not conducive to the use of intensive farming in agriculture, resulting in a decline in land productivity [63] and an increase in agricultural carbon intensity. The direct effect coefficient of agricultural disaster rate on agricultural carbon intensity is 0.003, and it is significant at the 1% level. For each unit of agricultural disaster rate increase, agricultural carbon intensity will increase by 0.003%. According to the research of He et al. [58], land damage will affect the final output, but the input of production factors in the early stage will not reduce agricultural carbon emissions, which will lead to an increase in agricultural carbon intensity. The direct effect coefficient of agricultural fiscal expenditure on agricultural carbon intensity is 0.037, and it is significant at the 1% level. For each unit of increase in agricultural disaster rate, agricultural carbon intensity will increase by 0.037%. The increase in agricultural financial expenditure will expand the scale of agricultural production, which is consistent with the research of Wang and Li [59]. This may be because agricultural fiscal expenditures have increased the input of production factors such as fertilizers and pesticides, thus increasing agricultural carbon intensity. The direct effects of other variables on agricultural carbon intensity were not significant.

From the perspective of indirect effects, the digital economy has a significant inhibitory effect on the agricultural carbon intensity of adjacent areas. For each unit of increase in the digital economy, the agricultural carbon intensity of adjacent areas will decrease by 0.596%. Zhou, Lan, Zhao, and Zhou [31] state that the positive spillover effect of the digital economy will promote the development of the digital economy in adjacent regions. As mentioned above, the digital economy can promote the dissemination of information between regions. The information here naturally also includes the development experience of the digital economy. When these experiences flow into adjacent areas, they can play a positive demonstration role, thereby reducing the agricultural carbon intensity of adjacent areas. The agricultural disaster rate has a significant role in promoting the agricultural carbon intensity of adjacent areas. For each unit of increase in the agricultural disaster rate, the agricultural carbon intensity of adjacent areas will increase by 0.008%. The ability of agriculture to resist natural disasters is very limited, and agricultural production is greatly affected by the natural environment. Geographically adjacent areas suffer from roughly the same natural disasters, so when a province’s agricultural disaster rate increases, the adjacent areas’ rates will also increase, thereby increasing agricultural carbon intensity. Agricultural fiscal expenditure has a significant role in promoting the agricultural carbon intensity of adjacent areas. For each unit of increase in agricultural fiscal expenditure, the agricultural carbon intensity of adjacent areas will increase by 0.168%. The competitive incentive mechanism among officials may make officials in adjacent regions imitate each other, so there will be a positive spatial spillover effect of agricultural financial support policies, leading to an increase in agricultural carbon intensity in adjacent regions. The indirect effects of other variables on agricultural carbon intensity were not significant.

In terms of total effect, the digital economy has a significant inhibitory effect on agricultural carbon intensity, the agricultural disaster rate and agricultural fiscal expenditure have a significant promoting effect on agricultural carbon intensity, and the total effect of other variables on agricultural carbon intensity is not significant.

## 5. Conclusions

Using the data of 30 provinces in mainland China from 2011 to 2019, this paper constructs a spatial Durbin model and a mediation effect model, and empirically examines the impact of the digital economy on agricultural carbon intensity. This paper also innovatively introduces agricultural technology progress to explore its mediating role in this effect. This paper aimed to find out how the digital economy affects the carbon intensity of agriculture in the China context and determine the mechanism behind this effect, in a bid to use the research conclusions to provide some reference for the Chinese government to reduce carbon emissions in agriculture. The research conclusions are as follows:

(1) China’s agricultural carbon intensity fluctuated and decreased from 2011 to 2019, and there has been agricultural carbon emission reduction. However, due to the large scale of production, the current agricultural carbon intensity is still very high. The digital economy has grown steadily year by year. The progress of agricultural technology is also on the rise, but the characteristic of “high energy consumption and high emissions” still exists, and the inhibitory effect on agricultural carbon intensity needs to be improved.
(2) The improvement of China’s digital economy will significantly reduce the carbon intensity of agriculture, and the advancement of agricultural technology has played an intermediary role in this impact.
(3) The improvement of China’s digital economy can significantly reduce the carbon intensity of agriculture in adjacent regions through spatial spillover effects.

## 6. Recommendations

Based on the above conclusions, the following policy recommendations are put forward:

(1) Based on the fact that the digital economy can effectively reduce the carbon intensity of agriculture, the government should increase investment in the internet industry and accelerate the implementation of 5G, artificial intelligence, and other internet technologies. The government should also accelerate the integration of digital economy and agriculture to build a data platform for agricultural production and promote the development of rural digital economy. At the same time, it is necessary to further develop digital inclusive finance, use the advantages of digital service channels, big data, cloud computing, and other technological methods to meet farmers’ financial needs, stimulate farmers’ innovation and entrepreneurship, and achieve high-quality agricultural development.
(2) The inhibitory effect of the digital economy on agricultural carbon intensity has a positive spatial spillover effect. The governments of neighboring provinces should break down administrative barriers, coordinate and cooperate with each other on internet infrastructure construction, agricultural technology innovation and application, and fully release the spatial contribution capacity of the digital economy to agricultural carbon reduction.
(3) The digital economy reduces the carbon intensity of agriculture by improving technological progress. Therefore, it is necessary to further increase investment in technological research and development in the agricultural field in the future, and focus on agricultural technologies with the characteristics of “improving productivity and controlling pollution,” thereby improving the carbon emission reduction capacity of agricultural technological progress. Second, an important premise for this mechanism to work is that farmers know how to use information technology and agricultural production technology. Therefore, the government should also improve farmers’ production skills and information equipment use skills through training. This will increase farmers’ human capital and create conditions for promoting and applying information technology and agricultural production technology in rural areas.

## 7. Deficiencies and Prospects

Based on the shortcomings of this study and the existing conclusions, follow-up research can start from the following aspects: (1) Due to the difficulty of data collection, this paper only takes 30 provinces in mainland China as the research object. Subsequent research can take prefecture-level cities as the research objects, and put forward more targeted policy suggestions based on their characteristics, and (2) Subsequent research can add the measurement of planting carbon sinks to obtain more accurate agricultural carbon emissions.

## Figures and Tables

**Table 1:** Carbon emissions coefficient.

Input Elements	Carbon Emission Coefficient	Reference Sources
Fertilizer	0.8956 kg C/kg	[36]
Pesticide	4.9341 kg C/kg	[36]
Agricultural film	5.18 kg C/kg	College of Resources and Environmental Sciences, Nanjing Agricultural University
Irrigation	266.48 kg C/hm^2^	[22]
Ploughing	16.47 kg C/hm^2^	[34]
Machinery	0.18 kg C/kW	[34]
Diesel oil	0.5927 kg C/kg	[22,39]

Note: kg C represents the mass of the carbon molecule.

**Table 2:** Digital economy comprehensive index system.

Subsystem	Indicators	Definition	Unit of Measurement	Weights
Internet	Internet penetration rate	Number of internet users per 100 people	-	0.220
	Internet-related employees	Proportion of employees in computer service and software industries in the unit employees	%	0.176
	Internet-related output	Total number of telecommunication services per capita	CNY	0.151
	The number of mobile internet users	Mobile phone users per 100 people	-	0.226
Digital finance	The digital inclusive financial index	The digital inclusive financial index	-	0.227

**Table 3:** Definition of all relevant variables used in the paper.

Symbol	Variable	Definition	Unit of Measurement
Explained variable			
ACI	Agricultural carbon intensity	Total agricultural carbon emissions/Value-added of primary industry	Ton/ten thousand Yuan
Explanatory variable			
DIG	Digital economy	Digital economy index	-
Mediating variable			
TE	Agricultural technological progress	Total number of invention patents and utility model patents in agriculture per year/Employees in the primary industry	items/10 thousand people
Control variable			
UR	Urbanization rate	Urban population/Total population	%
ER	Environmental regulation	Environmental pollution control investment/GDP	%
STRU	Industrial structure	Value-added of non-agricultural industrial/GDP	%
RTI	Road traffic infrastructure	Road and rail mileage per unit area in each province	10 thousand kilometers
AFFI	Agricultural disaster rate	Land affected area/Total sown area	%
AFE	Agricultural fiscal expenditure	Fiscal expenditure on agriculture, forestry and water affairs/Total expenditure on government fiscal final accounts	%

**Table 4:** Descriptive statistics for the variables.

Variables	N	Mean	Std. Deviation	Min	Max
ACI	270	0.223	0.084	0.101	0.508
DIG	270	0.296	0.161	0.020	0.815
TE	270	6.161	11.766	0.139	76.386
UR	270	57.636	12.178	35.000	89.600
STRU	270	90.255	5.132	73.800	99.700
ER	270	1.472	0. 796	0.300	4.841
RTI	270	14.942	7.865	1.208	33.709
AFFI	270	15.403	0.796	0.300	4.841
AFE	270	11.397	3.189	4.110	18.966

**Table 5:** Benchmark regression and mechanism test results of digital economy influencing agricultural carbon intensity.

	ACI		TE	ACI
	(1)	(2)	(3)	(4)
DIG	−0.243 ***	−0.250 ***	43.495 ***	−0.134 **
TE				−0.003 ***
LnUR		0.061	−77.244 ***	−0.146 ***
LnER		0.005	0.834	0.007
LnSTRI		0.618 ***	6.479	0.636 ***
LnRTI		0.001	−24.389 ***	−0.064 **
LnAFFI		0.003 **	−0.437 **	0.002 **
LnAFE		0.047 ***	−3.204	0.039 ***
_cons	0.257 ***	−2.885 ***	339.734 ***	−1.975 **
Year fixed	YES	YES	YES	YES
Province fixed	YES	YES	YES	YES
Observations	270	270	270	270
R^2^	0.153	0.304	0.584	0.445

Note: ** and *** indicate that the estimated coefficients passed the Z-test at the 5% and 1% levels of significance, respectively.

**Table 6:** Global Moran’s I of agricultural carbon intensity in China from 2011 to 2019.

Year	Moran’s Index	Z-Statistics	*p*-Value	Year	Moran’s Index	Z-Statistics	*p*-Value
2011	0.041	0.792	0.214	2016	0.088	1.299	0.097
2012	0.051	0.894	0.186	2017	0.092	1.340	0.090
2013	0.007	0.431	0.333	2018	0.086	1.281	0.100
2014	0.022	0.603	0.273	2019	0.095	1.402	0.080
2015	0.054	0.947	0.172				

**Table 7:** LM test, Wald test, Hausman test, and LR test results.

Variable	W
	Chi2-Statistic
LM-LAG	193.282 ***
Robust LM-LAG	16.870 ***
LM-ERR	179.655 ***
Robust LM-ERR	3.242 *
Wald-SAR	53.110 ***
Wald-SEM	41.190 ***
LR-SAR	47.870 ***
LR-SEM	39.620 ***
Hausman	12.390 *

Note: * and *** indicate that the estimated coefficients passed the Z-test at the 10% and 1% levels of significance, respectively.

**Table 8:** Spatial Durbin Model estimation and test results.

Variable	SDM	Variable	SDM
DIG	−0.174 ***	W * LnUR	0.117
LnUR	0.004	W * LnER	0.007
LnER	0.006	W * LnSTUR	−1.167 ***
LnSTUR	0.840 ***	W * LnRTI	0.140
LnRTI	−0.011	W * LnAFFI	0.004 **
LnAFFI	0.003 ***	W * LnAFE	0.100 ***
LnAFE	0.030 **	ρ	0.363 ***
W * DIG	−0.329 **	Log-likelihood	736.460

Note: ** and *** indicate that the estimated coefficients passed the Z-test at the 5% and 1% levels of significance, respectively.

**Table 9:** Direct effect, indirect effect, and total effect of factors affecting agricultural carbon intensity.

Variable	Direct Effect	Indirect Effect	Total Effect
DIG	−0.200 ***	−0. 596 ***	−0.796 ***
LnUR	0.011	0.188	0.199
LnER	0.007	0.015	0.022
LnSTUR	0.777 ***	−1.319 ***	−0.542
LnRTI	−0.001	0.205	0.203
LnAFFI	0.003 ***	0.008 ***	0.011 ***
LnAFE	0.037 ***	0.168 ***	0.205 ***

Note: *** indicate that the estimated coefficients passed the Z-test at the 1% levels of significance.

## Data Availability

The data presented in this paper are available on request from the corresponding author.

## Appendix A

**Table A1:** Chinese provincial names and corresponding abbreviations.

Province								
Shanghai (SH)	Jiangsu (JS)	Zhejiang (ZJ)	Anhui (AH)	Fujian (FJ)	Jiangxi (JX)	Shandong (SD)	Taiwan (TW)	Beijing (BJ)
Tianjin (TJ)	Shanxi (SX)	Hebei (HE)	Inner Mongoria (IM)	Henan (HA)	Hubei (HB)	Hunan (HN)	Guangdong (GD)	Hainan (HI)
Guangxi (GX)	Hong Kong (HK)	Macao (MO)	Chongqing (CQ)	Sichuan (SC)	Guizhou (GZ)	Yunnan (YN)	Tibet (XZ)	Shaanxi (SN)
Gansu (GS)	Qinghai (QH)	Ningxia (NX)	Xinjiang (XJ)	Heilongjiang (HL)	Jilin (JL)	Liaoning (LN)		

**Table A2:** Regional division of grain in China.

Areas	Province			
Main grain producing areas	Liaoning	Inner Mongoria	Henan	Heilongjiang
	Hebei	Jiangxi	Hubei	
	Shandong	Hunan	Jiangsu	
	Jilin	Sichuan	Anhui	
Main grain sales areas	Beijing	Zhejiang	Hainan	Guangdong
	Tianjin	Fujian	Shanghai	
Grain production and sales balance areas	Hainan	Shaanxi	Xinjiang	Ningxia
	Chongqing	Gansu	Shanxi	
	Yunnan	Qinghai	Guizhou	
